# Influence of adjuvant chemotherapy on survival for patients with completely resected high-risk stage IB NSCLC

**DOI:** 10.1186/s13019-023-02457-1

**Published:** 2024-01-02

**Authors:** Zi-Qing Shen, Kun-Peng Feng, Zi-Yao Fang, Tian Xia, Shu Pan, Cheng Ding, Chun Xu, Sheng Ju, Jun Chen, Chang Li, Jun Zhao

**Affiliations:** Department of Thoracic Surgery, The First Affiliated Hospital of Soochow University, Medical College of Soochow University, Suzhou, 215000 China

**Keywords:** Adjuvant chemotherapy, Early stage, Non-small cell lung cancer, High risk

## Abstract

**Background:**

The use of adjuvant chemotherapy (ACT) in completely resected stage IB NSCLC is still controversial. This study aims to investigate the efficacy of ACT in pathological stage IB non-small cell lung cancer (NSCLC) with high risk factors.

**Methods:**

Patients with pT2aN0M0 stage IB NSCLC who underwent complete resection from 2013 to 2017 were retrospectively analyzed. Univariate and multivariable logistic regression analysis was used to assess potential independent risk factors associated with poor prognosis. To compare survival between patients who received ACT and those who did not.

**Results:**

In univariate and multivariate analyses, adenocarcinomas with predominantly micropapillary (MIP) and solid patterns (SOL), poorly differentiated squamous cell carcinoma (SCC), number of lymph nodes dissected less than 16 and tumor size larger than 36 mm were identified as high-risk factors for recurrence. In patients with high risk factors for recurrence, ACT resulted in significantly longer DFS (HR, 0.4689, 95%CI, 1.193–3.818; p = 0.0108) and OS (HR, 0.4696, 95%CI, 0.6578–6.895; p = 0.2073), although OS failed to reach statistically significance. After propensity score matching (PSM), 67 pairs of patients were 1:1 matched in the two groups and all baseline characteristics were well balanced. The results also demonstrated that ACT was associated with improved DFS (HR, 0.4776, 95%CI, 0.9779–4.484; p = 0.0440) while OS was not significantly different (92.5% vs. 91.0%; HR, 0.6167, 95%CI, 0.1688–2.038; p = 0.7458). In patients with low-risk factors for recurrence, DFS (HR, 0.4831, 95%CI, 0.03025-7.715; p = 0.6068) and OS (HR, 0.969, 95%CI, 0.08364-11.21; p = 0.9794) was not significantly different between those who received ACT and those who did not.

**Conclusion:**

In patients with completely resected stage IB NSCLC, ACT can improve survival in patients with high risk for recurrence. Further large multicenter studies are needed to confirm these findings.

## Introduction

Lung cancer ranks as one of the most common malignancies worldwide and is the most frequent cancer and cause of cancer death in men and women combined [1, 2]. Surgical resection is the main treatment of choice for early-diagnosed lung cancers [3]. Postoperative recurrence is a major factor in poor postoperative prognosis and can be controlled with ACT in patients with resected stage II and IIIA NSCLC [4–7]. However, ACT use for stage IB NSCLC remains controversial [8, 9]. There are many guidelines for NSCLC precise therapies. The commonly used clinical guidelines include the National Comprehensive Cancer Network (NCCN) guideline, the Japanese Society of Lung Cancer guideline, European Society of Cancer Internal Medicine (ESMO) guideline, and Chinese Society of Clinical Oncology (CSCO) guideline. According to the latest NCCN guideline, postoperative chemotherapy is not required as a routine treatment for patients with stage IB, but it is recommended for patients with high-risk factors (including tumor size＞4 cm, visceral pleural invasion, lymph-vascular invasion, poor tumor differentiation, wedge resection, and incomplete lymph node sampling) in stage IB [10]. In contrast, the latest CSCO guidelines in China consider that stage IB NSCLC (including lung cancer with high-risk factors) generally does not recommend adjuvant chemotherapy due to the lack of high-level evidence to support it. The ESMO clinical practice guidelines also do not recommend postoperative chemotherapy for stage IB patients [11]. The diagnosis and treatment guidelines formulated by the Japanese Society of Lung Cancer recommends the use of oral tegafur-uracil for patients with stage IA/IB/IIA with a total tumor size > 2 cm after complete resection. The Cancer and Leukemia Group B (CALGB) 9633 trial is the first multi-institutional randomized controlled trial (RCT) designed specifically for stage IB NSCLC [12]. Results of this RCT found that no significant survival advantage was observed for the entire cohort and adjuvant chemotherapy should not be considered the standard of care for stage IB NSCLC. Only a post-hoc subgroup analysis demonstrated a significant survival difference in favor of ACT for patients who had tumors ≥ 4 cm in diameter. However, a mass of this size is classified as stage IIA according to the Eighth Edition of the Lung Cancer Tumor-lymph Node Metastasis (TNM) staging system [13]. In recent years, there have been many studies on whether patients with stage IB NSCLC should receive adjuvant therapy, but there is still no consensus [9, 14–21].

The purpose of our study was to further analyze and evaluate the role of platinum-based doublet ACT in patients with NSCLC by collecting and analyzing patients with stage IB (T2aN0M0) who underwent complete surgical resection from 2013 to 2017 in the First Affiliated Hospital of Soochow University.

## Patients and methods

### Patients

**Fig. 1:**
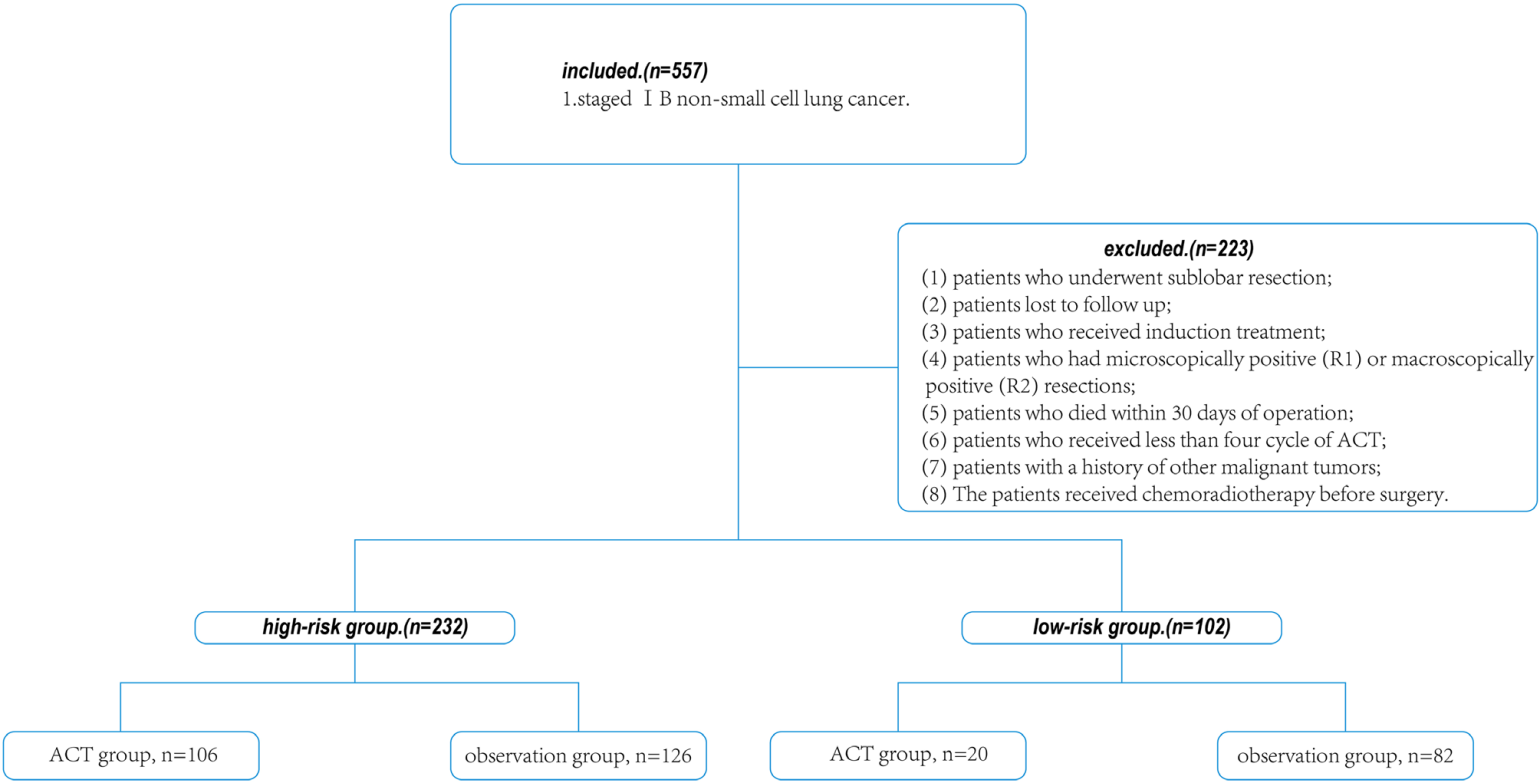
Flow diagram of patients studied

This retrospective study was approved by the Institutional Review Board of the First Affiliated Hospital of Soochow University (ethical approval No.2,022,279). A total of 334 patients with stage 1b (T2aN0M0, the maximum diameter of primary tumor > 3 cm, ≤ 4 cm; Or with any of the following conditions: involving the main bronchus but not reaching the talar carina; Visceral pleura was involved; With partial or whole lung, pneumonia and atelectasis, no regional lymph node metastasis, no distant metastasis) NSCLC who had undergone VATS(video-assisted thoracoscopic surgery) curative R0 resections between 2013 January and 2017 July were reviewed. NSCLC staging was performed according to the 8th edition of the TNM classification [13]. The inclusion criteria were [1] patients with primary non-metastatic NSCLC; [2] patients who received radical resection for lung cancer as the first step of treatment, without opting for other treatments such as preoperative radiotherapy or chemotherapy; [3] postoperative histopathological diagnosis was confirmed as NSCLC. Exclusion criteria were as follows: [1] patients who underwent sublobar resection [2] patients lost to follow up; [3] patients who received induction treatment; [4] patients who had microscopically positive (R1) or macroscopically positive (R2) resections; [5] patients who died within 30 days of operation; [6] patients who received less than four cycle of ACT [7] patients with a history of other malignant tumors [8] The patients received chemoradiotherapy before surgery (Fig. 1).

All patients received standardized preoperative examinations, including head and chest computed tomography (CT), abdominal ultrasound, lower extremity vascular and carotid ultrasound, echocardiography and electrocardiogram and lung function. Some patients also underwent radionuclide bone scans and positron emission tomography (PET/CT) prior to surgery. The surgical specimens were formalin-fixed, dehydrated, paraffin-embedded, sliced at a thickness of 5 μm, and stained with hematoxylin and eosin. Histopathological analysis of each sample was performed by an experienced pulmonary pathologist and verified by another senior pulmonary pathologist. ACT was defined as postoperative chemotherapy within 2–6 weeks of surgery and the need for more than 4 courses of treatment. The drugs used were determined by oncologists and were all platinum-based dual-drug regimens. Routine follow-up after completion of treatment included outpatient clinic visits every 3 months for the first 2 years and every 6 months thereafter. The contents of each follow-up examination included physical examination, chest CT, and tumor marker detection. For patients who failed to come to the outpatient clinic for re-examination, our department also conducted telephone follow-up and recorded the patient’s condition. When any symptoms or signs of recurrence are observed, further testing of the patient is completed immediately. For example, when the patient has unpleasant symptoms such as headache, dizziness, it is recommended that the patient should undergo a brain Magnetic Resonance Imaging (MRI) or CT scan. When the patient has bone pain, it is recommended that the patient should undergo a bone scan.

### Statistical analysis

Continuous variables were compared by using the *t*-test and categorical variables were compared by using the χ² test. The duration of overall survival (OS) was defined as the interval between the date of surgical resection and the date of death from any cause. Disease free survival (DFS) was defined as the interval between the date of surgical resection and the date of the first event locoregional or distant recurrence or death from any cause. Univariate and multivariable logistic regression analysis was used to assess potential independent factors associated with DFS and OS. The Kaplan-Meier method with log-rank test was performed to compare the survival curves for DFS and OS. Considering the existence of bias in the analysis and control process, we used PSM to reduce the potential effects of bias. A logistic regression model was established to calculate the propensity score based on the following covariates: age, sex, tumor size, histologic type and grade, visceral pleural invasion (VPI), lympho-vascular invasion, surgical procedures and the number of lymph nodes dissected. Patients who received ACT were matched with patients who underwent surgery only by a 1:1 greedy algorithm without replacement.

Statistical analyses were performed using SPSS (SPSS 26.0 for Windows, SPSS). All the statistical tests were two-sided and p values of 0.05 or less were considered statistically significant.

## Results

The clinical date of the 334 patients in this study are summarized in Table 1. The median follow-up period after surgery was 30.0 months. During this period, tumor recurrence was observed in 39 patients (11.67%) and 15 patients (4.2%) died of lung cancer progression. Of the 334 patients, 126 patients received ACT and 208 did not.

**Table 1:** Patients’ characteristics

	N = 334 (100%)
Age (years)	63.27545 ± 9.266274
**Gender**	
Female	158(47.30%)
Male	176(52.69%)
**Histology**	
Adenocarcinoma	278(83.2%)
LEP	37(11.0%)
ACI-PAP	186(55.6%)
MIP-SOL	55(16.4%)
Squamous cell carcinoma	45(13.4%)
Poor differentiated	24(7.1%)
Moderate/high differentiated	21(6.2%)
Mixed	11(3.2%)
Number of resected lymph nodes	17.43413 ± 6.823394
Tumor diameter(mm)	32.61677 ± 3.625463
**Vascular invasion**	
Yes	5(1.6%)
No	329(98.3%)
**Visceral pleural invasion**	
Yes	124(37.12%)
No	210(62.87%)
**Adjuvant chemotherapy**	
Yes	126(37.72%)
No	208(62.27%)
**Recurrence**	
Yes	39(11.67%)
No	295(88.32%)
**Death**	
Yes	15(4.2%)
No	319(95.7%)

**Fig. 2:**
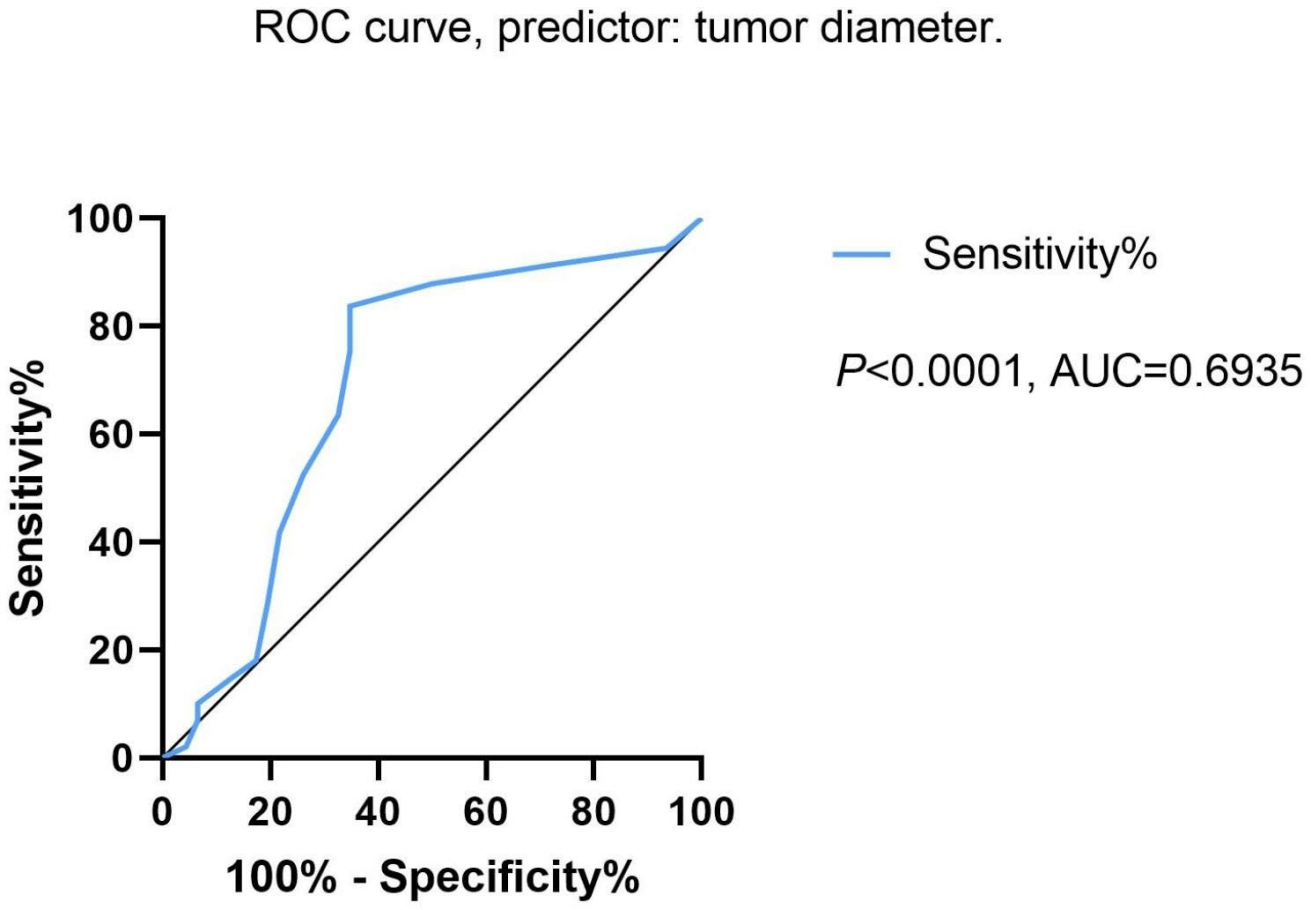
ROC curve, predictor: tumor diameter

Because the number of deaths was too small (only 15 cases) and all of them were caused by tumor recurrence, the results of the risk factor analysis for the outcome of death were not statistically significant. Therefore, we performed the further studies using the results of risk factor analysis using tumor recurrence as the outcome. The results of univariate analysis showed that adenocarcinomas with predominantly MIP and SOL patterns, poorly differentiated SCC, number of lymph nodes dissected less than 16 and the tumor size were risk factors for tumor recurrence. However, multivariate analysis showed that in addition to the above factors, VPI was also a risk factor for tumor recurrence. The results are shown in Table 2. Although VPI was not statistically significant in the univariate logistic regression analysis, it was found to be statistically significant in the multivariate logistic regression analysis. VPI may be as a synergistic factor of several other risk factors in the process of tumor recurrence. Since the tumor size is a continuous variable, we decided to use the ROC curve to obtain a diagnostic cut-off point. The results of the ROC curve suggested that the tumor diameter greater than 36 mm was a risk factor for tumor recurrence (Fig. 2).

**Table 2:** Univariable and multivariable analyses of DFS and OS

Variable	Univariable analyses			Multivariable analyses			
	HR	95%CI	*P* value	HR	95%CI		*P* value
**disease-free survival (DFS)**							
Age	1.036	0.998 ~ 1.075	0.066	1.001	0.959 ~ 1.045	0.967	
Male sex	1.326	0.705 ~ 2.491	0.381	0.936	0.427 ~ 2.055	0.87	
ACI-PAP							
LEP	1.271	0.2596.243	0.767	1.025	0.201 ~ 5.232	0.976	
MIP-SOL	12.714	5.188 ~ 31.161	< 0.001***	17.178	6.164 ~ 47.876	< 0.001***	
Poor differentiated SCC	18.827	6.453 ~ 54.928	< 0.001***	18.194	5.485 ~ 60.351	< 0.001***	
Moderate/high differentiated SCC	3.708	0.903 ~ 15.230	0.069	2.757	0.616 ~ 12.334	0.185	
Mixed	4.944	0.914 ~ 26.743	0.063	2.694	0.418 ~ 17.360	0.297	
Number of resected lymph nodes < 16	0.943	0.900 ~ 0.988	0.013*	0.904	0.851 ~ 0.961	< 0.001***	
Tumor diameter(mm)	1.222	1.108 ~ 1.348	< 0.001***	1.237	1.0981.394	< 0.001***	
Visceral pleural invasion	1.851	0.989 ~ 3.465	0.054	2.340	1.0635.155	0.035*	
ACT	0.932	0.475 ~ 1.831	0.838	0.568	0.249 ~ 1.296	0.179	
**Overall survival (OS)**							
Age	1.071	0.994 ~ 1.153	0.072	1.061	0.975 ~ 1.155	0.172	
Male sex	1.833	0.541 ~ 6.210	0.330	1.315	0.306 ~ 5.657	0.713	
ACI-PAP							
LEP	< 0.001	< 0.001	0.998	< 0.001	< 0.001	0.998	
MIP-SOL	5.571	1.513 ~ 20.523	0.010*	4.872	1.087 ~ 21.836	0.039*	
Poor differentiated SCC	4.136	0.716 ~ 23.901	0.113	2.864	0.391 ~ 20.999	0.0301*	
Moderate/high differentiated SCC	< 0.001	< 0.001	0.998	< 0.001	< 0.001	0.998	
Mixed	< 0.001	< 0.001	0.999	< 0.001	< 0.001	0.999	
Number of resected lymph nodes < 16	1.065	0.972 ~ 1.167	0.177	1.061	0.967 ~ 1.163	0.209	
Tumor diameter(mm)	1.301	1.073 ~ 1.577	0.008**	1.355	1.096 ~ 1.676	0.005**	
Visceral pleural invasion	3.552	1.047 ~ 12.050	0.042*	5.065	1.266 ~ 20.259	0.022*	
ACT	1.563	0.484 ~ 5.044	0.455	1.176	0.296 ~ 4.666	0.818	

HR, hazard ratio; CI, confidence interval

Based on the above results, we decided to use adenocarcinomas with predominantly MIP and SOL patterns, poorly differentiated SCC, number of lymph nodes dissected less than 16 and the tumor diameter greater than 36 mm as risk factors for tumor recurrence. We use risk factors for tumor recurrence to stratify patients into high-risk and low-risk groups. Patients with one or more risk factors for recurrence were included in the high-risk group, while patients without risk factors for recurrence were included in the low-risk group. According to this standard, a total of 232 patients were included in the high-risk tumor group, including 106 patients who received ACT and 126 patients who did not receive. After PSM, 67 pairs of patients were 1:1 matched in the two groups and all baseline characteristics were well-balanced. Details of each confounding variable before and after matching are summarized in Table 3. And a total of 102 patients were included in the low-risk tumor group, including 20 patients who received ACT and 82 patients who did not receive. The results are shown in Table 4.

**Table 3:** Clinicopathological characteristics of patients with high risk before and after PSM

Characterisitc	Before PSM			After PSM		
	ACT group (n = 106)	Observation group (n = 126)	P value	ACT group (n = 67)	Observation group (n = 67)	P value
Age	64.16 ± 8.17	64.67 ± 9.128	0.659	65.2090 ± 7.83840	64.8358 ± 8.82420	0.796
**Gender**			< 0.001***			0.287
Male	83(78.30%)	70(55.56%)		44(65.67%)	38(56.71%)	
Female	23(21.70%)	56(44.44%)		23(34.32%)	29(43.28%)	
**Histologic type**						
Adenocarcinoma	83(78.30%)	100(79.3%)		50(74.62%)	53(79.10%)	
LEP	10(9.43%)	20(15.87%)		7(10.44%)	12(17.91%)	
ACI-PAP	53(50.00%)	45(35.71%)		23(34.32%)	21(29.85%)	
MIP-SOL	20(18.87%)	35(27.78%)		20(29.85%)	20(29.85%)	
Squamous cell carcinoma	19(17.92%)	22(17.46%)		14(20.89%)	12(17.91%)	
Poor differentiated	10(9.43%)	14(11.11%)		10(14.92%)	7(10.44%)	
Moderate/high differentiated	9(8.49%)	8(6.35%)		4(5.97%)	5(7.46%)	
Mixed	4(3.77%)	4(3.17%)		3(4.47%)	2(2.98%)	
Number of resected lymph nodes	15.1 ± 5.224	15.59 ± 8.779	0.600	15.5522 ± 7.52226	16.0597 ± 6.91700	0.685
Tumor diameter(mm)	33.44 ± 3.532	33.12 ± 3.969	0.515	33.1642 ± 3.64566	33.5075 ± 3.97101	0.603
**Vascular invasion**			0.660			0.315
Yes	3(2.8%)	2(1.5%)		1(1.4%)	0(0%)	
No	103(97.1%)	124(98.4%)		66(98.5%)	67(100%)	
**Visceral pleural invasion**			0.019*			0.723
Yes	29(27.3%)	54(42.8%)		27(40.2%)	25(37.3%)	
No	77(72.6%)	72(57.1%)		40(59.7%)	42(62.6%)	
**Recurrence**			0.021*			0.042*
Yes	14(13.2%)	32(25.3%)		9(13.4%)	18(26.8%)	
No	92(86.7%)	94(74.6%)		58(86.5%)	49(71.6%)	
**Death**			0.774			0.628
Yes	5(4.7%)	7(5.5%)		5(7.4%)	6(8.9%)	
No	101(95.2%)	119(94.4%)		62(92.5%)	61(91.0%)	

**Table 4:** Clinicopathological characteristics of patients with low risk

	ACT group (n = 20)	observation group (n = 82)	*P* value
Age	60.90 ± 12.42197	6 ± 9.42682	0.806
**Gender**			0.036*
Male	1(5%)	22(26.82%)	
Female	19(95%)	60(73.17%)	
**Histology**			
Adenocarcinoma	18(90%)	77(93.90%)	
LEP	2(10%)	5(6.09%)	
ACI-PAP	16(80%)	72(87.80%)	
MIP-SOL	0(0%)	0(0%)	
Squamous cell carcinoma	2(10%)	2(2.43%)	
Poor differentiated	0(0%)	0(0%)	
Moderate-high differentiated	2(10%)	2(2.43%)	
Mixed	0(0%)	3(3.65%)	
Number of resected lymph nodes	21.23 ± 5.224	22.59 ± 8.779	0.623
Tumor diameter(mm)	32.20 ± 2.52566	30.87 ± 2.76839	0.572
**Vascular invasion**			0.996
Yes	0(0%)	2(2.43%)	
No	20(100%)	80(97.56%)	
**Visceral pleural invasion**			0.984
Yes	8(40%)	33(40.24%)	
No	12(60%)	49(59.75%)	
**Recurrence**			0.543
Yes	1(5%)	2(2.43%)	
No	19(95%)	80(97.56%)	
**Death**			0.543
Yes	1(5%)	2(2.43%)	
No	19(95%)	80(97.56%)	

**Fig. 3:**
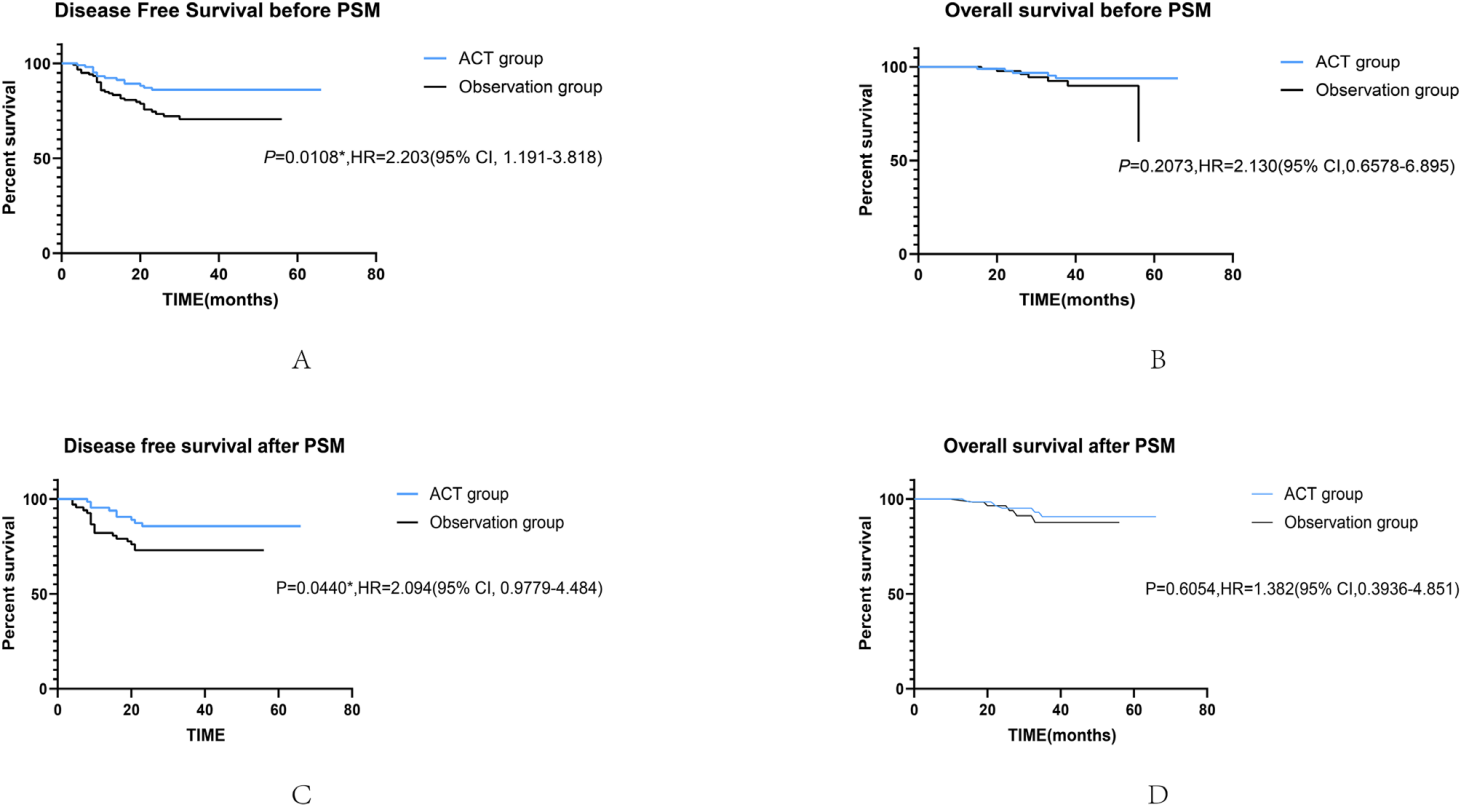
Kaplan-Meier survival curves for DFS (**A**) and OS (**B**) in high-risk patients with or without adjuvant chemotherapy before PSM. Kaplan-Meier survival curves of DFS (**C**) and OS (**D**) in high-risk patients with or without adjuvant chemotherapy after PSM

**Fig. 4:**
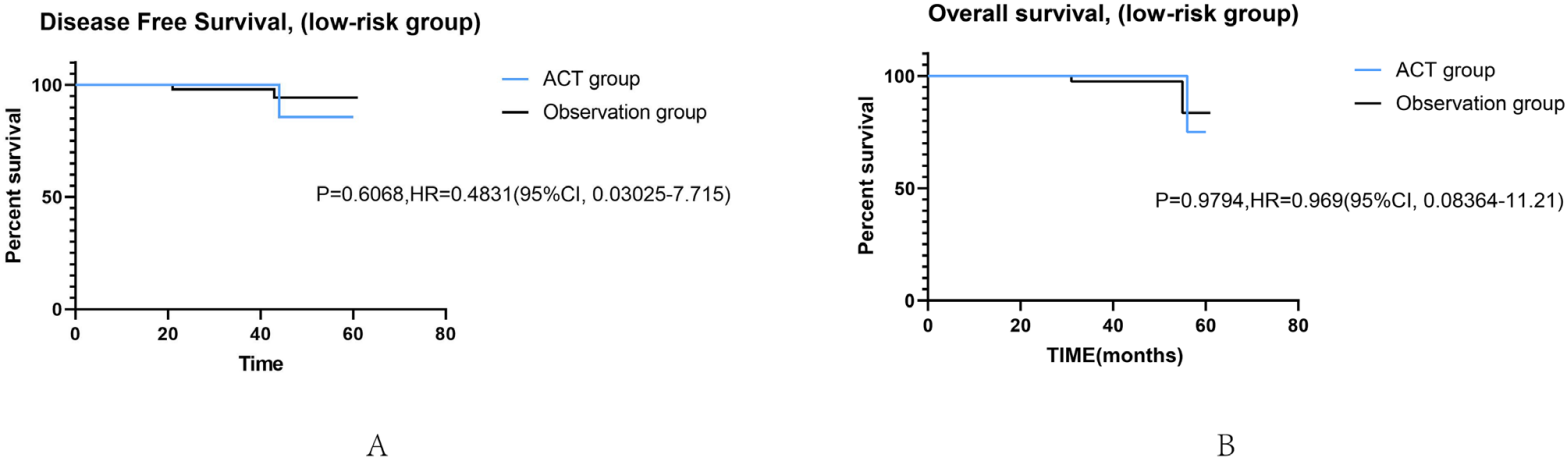
Kaplan-Meier survival curves for DFS (**A**) and OS (**B**) in high-risk patients with or without adjuvant chemotherapy before PSM

Survival curves were used to evaluate whether postoperative ACT was beneficial for patients with high-risk factors. With DFS (HR, 0.4689, 95%CI, 1.193–3.818; p = 0.0108) as the outcome, the survival curve of patients receiving ACT was significantly better than those who did not, while there was no significant difference in OS (HR, 0.4696, 95%CI, 0.6578–6.895; p = 0.2073) between the two groups (Fig. 3). After PSM in high-risk groups, ACT was associated with improved DFS (HR, 0.4776, 95%CI, 0.9779–4.484; p = 0.0440; Fig. 3) either. Similarly, there was no significant difference in OS (HR, 0.6167, 95%CI, 0.1688–2.038; p = 0.7458). In the low-risk group, there were no significant differences in DFS (HR, 0.6068, 95%CI, 0.03025-7.715; p = 0.4831) and OS (HR, 0.9794, 95%CI, 0.08364-11.21; p = 0.9794) between patients who received adjuvant chemotherapy and those who did not (Fig. 4).

## Discussion

Although ACT is the standard therapy for NSCLC in stage II–IIIA [7, 22–24], it is still controversial whether ACT improves survival in patients with stage IB NSCLC [9, 14–21]. In this study, we summarize the clinical data of patients with stage IB NSCLC who underwent complete surgical resection in our hospital from January 2013 to June 2017 to investigate the benefit of ACT in completely resected stage IB NSCLC. Based on the results of univariate and multivariable analysis, we identified patients with pathologic stage IB NSCLC that was at high risk for recurrence using tumor size larger than 36 mm, adenocarcinomas with predominantly MIP and SOL patterns, poorly differentiated SCC, number of lymph nodes dissected less than 16. For patients in the low-risk group, the survival period was relatively long regardless of chemotherapy. In contrast, in high-risk patients, the use of ACT improved DFS and OS. Although OS failed to reach statistical significance, this may be there were no sufficient death cases deaths in this study, and further studies are needed to expand the sample size in the future. These results suggest that our definitions of low-and high-risk groups was appropriate and that this grouping may be useful for decision-making in ACT for stage IB NSCLC.

Several studies have shown that tumor size predicts prognosis in patients with NSCLC after surgical resection [25–29]. Consistent with most previous studies, our findings suggest that patients with stage IB NSCLC with larger tumors (36 mm < tumor ≤ 40 mm) had significantly worse prognosis than those with smaller tumors. The first multi-institutional RCT study [12] designed specifically for stage IB NSCLC concluded that ACT can improve outcomes in patients with tumors ≥ 40 mm. However, according to the latest NSCLC TNM staging criteria [13], this population has been classified as category IIA patients. Our findings suggest that ACT can improve the prognosis of patients with stage IB lung cancer with a tumor size larger than 36 mm. For stage IB patients, it is still questionable whether to accept ACT therapy based solely on the size of the tumor. In clinical diagnosis and treatment, poorly differentiated NSCLC often progresses faster and has a worse prognosis than well and moderately differentiated tumors [30]. According to our univariate and multivariate outcome analysis, adenocarcinomas with predominantly MIP and SOL patterns, poorly differentiated SCC had a poor prognostic outcome. The World Health Organization (WHO) updated the pathological classification of lung tumors in 2015 and reclassified lung adenocarcinoma, dividing mixed adenocarcinoma into several subtypes including lepidic (LEP), acinar (ACN), papillary (PAP), MIP and SOL tumors [31]. Due to the small number of patients in some of the five subtypes and with reference to relevant cutting-edge research, we divided lung adenocarcinoma patients into three categories: LEP, ACN/PAP, and MIP/SOL [32]. In a report related to the Lung Assisted Cisplatin Evaluation (LACEBIO) study, patients with MIP/SOL adenocarcinoma (but not ACN/PAP adenocarcinoma) achieved DFS and specific DFS, but OS did not benefit from ACT [32, 33]. Hung et al. [15] found that ACT was a favorable prognostic factor for recurrence-free survival in patients with stage IB lung adenocarcinoma, especially in patients with a MIP/SOL pattern. In our study, we found that the MIP/SOL group had worse OS and DFS than LEP and ACN/PAP groups and ACT could improve the prognosis of these patients. The MIP/SOL pattern may be considered a stratification factor in the design of future clinical trials of ACT in patients with stage IB NSCLC. Furthermore, for patients with early-stage SCC, evidence from previous studies suggests that early-stage SCC may be associated with a higher probability of recurrence compared with patients with adenocarcinoma [34–36]. In addition to the relatively high risk of SCC recurrence, related studies also show that SCC histology is better than adenocarcinoma histology in predicting the efficacy of chemotherapy, which may partly explain the efficacy of ACT in patients with stage IB SCC [37, 38]. Xu et al. [16] demonstrated that ACT can improve the survival rate of patients with surgically resected stage IB lung SCC. We also concluded that ACT is beneficial to the prognosis of poorly differentiated SCC, but there was no statistical significance. It may be because the vast majority of SCC patients in our study were poorly differentiated SCC, and the number of patients with moderately and well-differentiated SCC was very small. Lymph node (LN) status is of great significance for the staging and prognosis of NSCLC. Extensive lymph node dissection can help detect occult lymph node metastasis and provide appropriate adjuvant therapy, thereby improving long-term survival. But at the same time, this step will increase the operation time and may lead to an increased probability of postoperative complications [39, 40]. Several studies have included the number of dissected LNs as an indicator of surgical quality and investigated the relationship between the number of dissected LNs and the prognosis of NSCLC [39–45]. Data from the ACOSOG Z0030 trial suggested that resection of 10 LNs might be considered adequate [39]. Similarly, the American College of Surgeons supports the removal of at least 10 lymph nodes as a quality indicator [46]. Liang et al. [40]proposed that in patients with stage I-IIIA resected NSCLC, more dissected LNs were associated with more accurate lymph node staging and better long-term survival. The study recommended 16 dissected LNs as the threshold for evaluating the quality of LN examination. For patients with stage IB NSCLC with poor LN evaluation, there is no clear recommendation for further adjuvant chemotherapy. According to the above cutting-edge research, we chose 16 lymph node dissections as the measurement standard when analyzing the risk factors affecting the prognosis of patients. To sum up, we chose 16 lymph node dissections as the measurement standard when analyzing the risk factors affecting the prognosis of patients. And according to univariate and multivariate logistic regression results, patients with less than 16 lymph node dissections had a worse prognosis, and chemotherapy could improve the prognosis of this population. The number of LNs examined at the time of NSCLC resection, to date, remains a forum of open discussion. VPI represents the ability of the tumor to penetrate the pleural elastin layer, indicating an elevated risk of seeding into the pleural cavity. The visceral pleura is rich in lymphatic vessels, and tumor cells eventually flow into the hilar lymph nodes [10, 17, 47]. Several studies have demonstrated that VPI is a major determinant of tumor stage and a risk factor for recurrence and poor survival [48, 49]. In the present study, multivariate regression analysis indicated that VPI stage IB NSCLC patients had worse prognosis, which was consistent with previous studies. Interestingly, univariate logistic regression did not show that VPI was a risk factor for tumor prognosis. We consider that VPI may be a synergistic factor for several other risk factors in the prognosis of stage IB NSCLC which is consistent with the results of some previous studies [50, 51]. These studies showed that VPI was a significant poor prognostic factor in node-negative patients with tumor size > 3 but ≤ 5 cm. Further multicenter studies are needed to confirm these findings.

Our study should be interpreted with caution because of several limitations. This study was retrospective in nature and single-center study, which may lead to patient selection bias and time trend bias. Another limitation is that the use of ACT and selection of the regimens used for adjuvant therapy were not randomized but were according to physician preference. In the future, multi-institutional studies and randomized clinical trials with large sample sizes are needed to further verify the value of ACT for the treatment of patients with stage IB NSCLC in the future.

## Conclusion

In conclusion, ACT improves survival in patients with stage IB NSCLC at high risk of recurrence. Further large multicenter larger sample size randomized clinical trials are needed to further identify the role of ACT and better systemic strategies need to been developed in resected stage IB NSCLC.

## Data Availability

The data supporting this study can be obtained from the corresponding author [Jun Zhao]; As the research data involve patient privacy and informed consent, the data will not be disclosed.

## Abbreviations

ACT: adjuvant chemotherapy
NSCLC: non-small cell lung cancer
LEP: lepidic
ACN: acinar
PAP: papillary
MIP: micropapillary
SOL: solid
SCC: squamous cell carcinoma
NCCN: National Comprehensive Cancer Network
ESMO: European Society of Cancer Internal Medicine
CSCO: Chinese Society of Clinical Oncology
RCT: randomized controlled trial
OS: overall survival
DFS: disease-free survival
PSM: propensity score matching
VPI: visceral pleural invasion
CT: computed tomography
PET/CT: positron emission tomography
MRI: Magnetic Resonance Imaging
LN: Lymph node
HR: hazard ratio
CI: confidence interval
